# Supplementary material for: The interactome of intact mitochondria by cross-linking mass spectrometry provides evidence for coexisting respiratory supercomplexes
Source: Mol Cell Proteomics. 2017 Dec 8;17(2):216–32. doi: 10.1074/mcp.RA117.000470 (PMC5795388; doi:10.1074/mcp.RA117.000470)
Supplement: Supplemental Data [file supp_RA117.000470_133922_0_supp_23978_fzffwf.zip › spectra_annotation/mito_spectra_annotation/131-1-18-1-11-1.pdf]

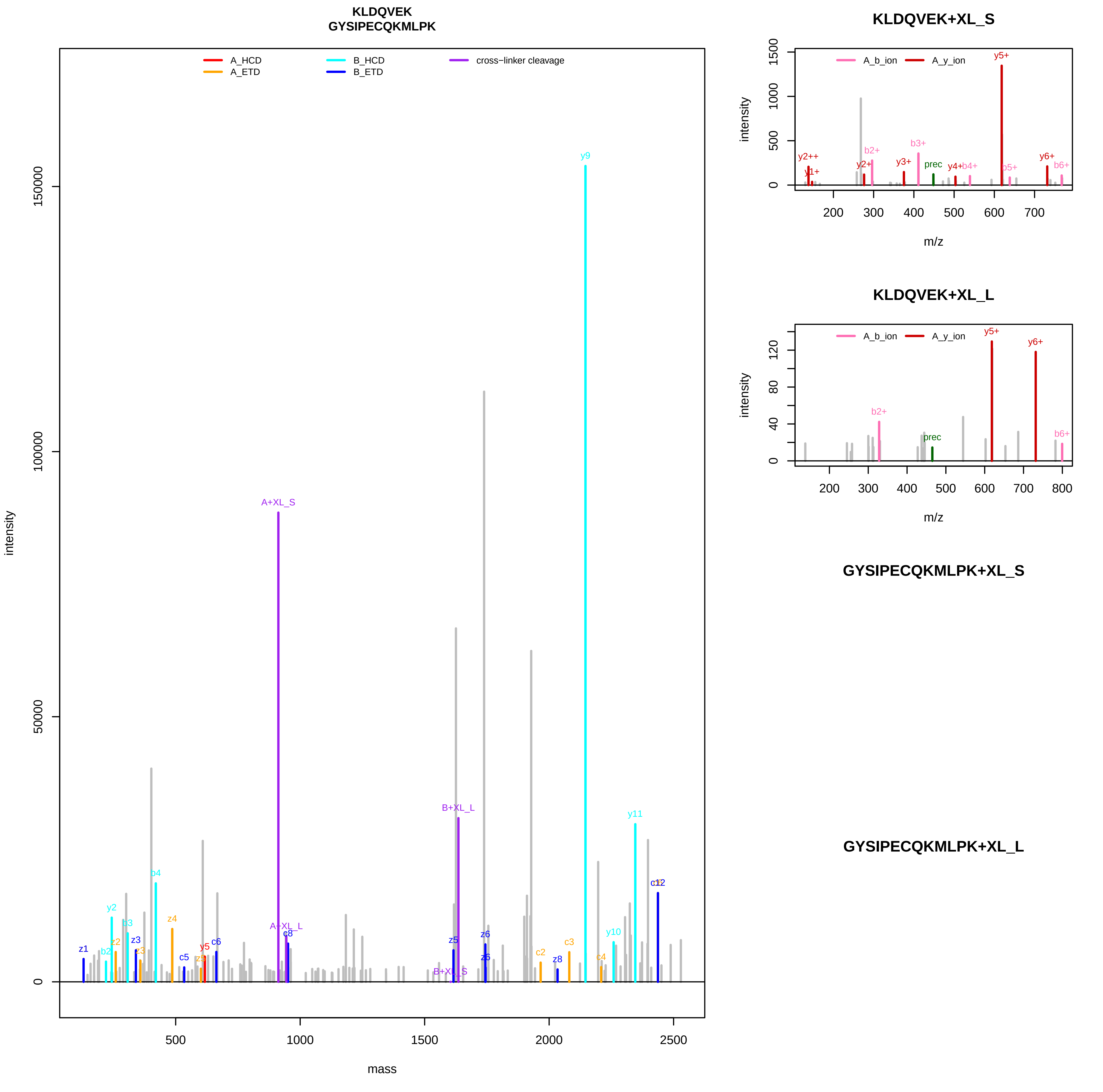
KLDQVEK
GYSIPECQKMLPK
A_HCD
A_ETD
B_HCD
B_ETD
cross−linker cleavage
intensity
mass
y9
A+XL_S
B+XL_L
A+XL_L
B+XL_S
y11
y10
c12
b4
y2
b3
b2
z1
z2
z3
z4
z5
z6
z8
c2
c3
c4
c5
c6
c8
y5
KLDQVEK+XL_S
A_b_ion
A_y_ion
y5+
y6+
b2+
b3+
b4+
b5+
b6+
y1+
y2+
y2++
y3+
y4+
prec
m/z
KLDQVEK+XL_L
GYSIPECQKMLPK+XL_S
GYSIPECQKMLPK+XL_L
